# Patterns of information literacy and their predictors among emergency department nurses: a latent profile analysis based on the person-context interaction theory

**DOI:** 10.1186/s12912-024-01756-9

**Published:** 2024-01-26

**Authors:** Chao Wu, Chunyan He, Jiaran Yan, Juan Du, Shizhe He, Zhaohua Ji, Yifei Wang, Hongjuan Lang

**Affiliations:** 1 Department of Nursing, Fourth Military Medical University, No.169 Changle West Road, Shaanxi, Shaanxi 710032 China; 2 Department of Nursing, Tongji Medical College, Huazhong University of Science and Technology, Hubei, China; 3 Department of Nursing, The Air Force Hospital of Northern Theater PLA, Shenyang, China; 4 Department of Epidemiology, Ministry of Education Key Lab of Hazard Assessment and Control in Special Operational Environment, School of Public Health, Fourth Military Medical University, No.169 Changle West Road, Shaanxi, Shaanxi 710032 China; 5 Department of Military Medical Psychology, Fourth Military Medical University, No.169 Changle West Road, Shaanxi, Shaanxi 710032 China

**Keywords:** Latent profile analysis, Emergency department nurses, Information literacy

## Abstract

**Background:**

With the development of information technology, information has been an important resource in clinical medicine, particularly within the emergency department. Given its role in patient rescue, the emergency department demands a high level of information literacy from nurses to effectively collect, analyze, and apply information due to the urgency and complexity of emergency nursing work. Although prior studies have investigated the information literacy of nursing staff, little has been undertaken in examining the patterns of information literacy and their predictors among emergency department nurses.

**Aim:**

To clarify the subtypes of information literacy among nurses in the emergency department and explore the factors affecting profile membership.

**Methods:**

A cross-sectional study was conducted among a convenience sample of 2490 nurses in the emergency department from April to June 2023. The clinical nurses completed the online self-report questionnaires including the general demographic questionnaire, information literacy scale, self-efficacy scale and social support scale. Data analyses involved the latent profile analysis, variance analysis, Chi-square tests and multivariate logistic regression.

**Results:**

Four latent profiles were identified: ‘Low information literacy (Class 1)’, ‘Moderate information knowledge (Class 2)’, ‘High information knowledge and support (Class 3)’ and ‘High information literacy (Class 4)’, accounting for 20.14%, 42.11%, 23.36% and 14.39%, respectively. Each profile displayed unique characteristics representative of different information literacy patterns. Age, years of work, place of residence, hospital grade, title, professional knowledge, using databases, reading medical literature, participating in information literacy training, self-efficacy, and social support significantly predicted information literacy profile membership.

**Conclusions:**

Information literacy exhibits different classification features among emergency department nurses, and over half of the nurses surveyed were at the lower or middle level. Identifying sociodemographic and internal-external predictors of profile membership can aid in developing targeted interventions tailored to the needs of emergency department nurses. Nursing managers should actively pay attention to nurses with low information literacy and provide support to improve their information literacy level.

**Relevance to clinical practice:**

Insights from the current study of the latent profile analysis are beneficial to hospital managers in understanding the different types of emergency department nurses’ information literacy. These insights serve as a reference for managers to enhance nurses’ information literacy levels.

**Supplementary Information:**

The online version contains supplementary material available at 10.1186/s12912-024-01756-9.

## Introduction

Along with the rapid development of computer applications and network communication technologies, we have entered an era of big data [1, 2]. Since big data has become a new strategic resource, its potential value and growth rate are changing the way of work, live, and think [3, 4]. Digital healthcare and Internet hospitals have become the main direction in healthcare development [5–7].

In the environment of digital intelligent medical, information literacy is a basic ability for nurses in clinical nursing practice [8]. Information literacy refers to the ability to perceive information needs, access information resources, and evaluate information values [9, 10]. Studies have found that good information literacy of nurses is conducive to promoting the health of patients and improving their survival outcomes [11, 12]. Besides, research has also shown that information capability is closely related to innovation behavior and work enthusiasm among nursing staff [13]. In clinical practice, nurses often encounter challenges when dealing with clinical problems, particularly in terms of locating the most relevant evidence identifying the right sources, using optimal search methods, and critically appraising the evidence [14].

The emergency department serves as the primary facility for managing severely ill patients during emergencies, therefore, the information management in the emergency department is especially important. As a special department for rescuing patients, it requires high information literacy for nurses to collect, process, and use information due to the urgency and complexity of emergency nursing work [15, 16]. Information literacy can help emergency department nurses make emergency care decisions and improve their work efficiency. Therefore, it is imperative to improve emergency department nurses’ information literacy and explore the patterns of information literacy and their predictors among emergency department nurses to help them improve the quality of emergency care.

The Person-Context interaction theory holds that the individuals and environment are not independent entities, but an integrated system. Individual behavior is predicted by both external situations and individual internal characteristics [17]. It can provide a theoretical framework to explore how variables such as individual factors and external factors affect emergency department nurses’ information literacy. Therefore, when exploring the information literacy of emergency nursing staff, we will use this theory as a guide to analyze from two aspects and more comprehensively explore the predictive factors of their information literacy.

## Measuring nurses’ information literacy

There are some studies on the information literacy of clinical nurses, but there is a notable lack of research specifically focused on the information literacy of emergency department nurses. Existing research showed that the level of information literacy among Chinese nursing staff was suboptimal due to factors such as education level and the state of medical development [18, 19], which impede their ability to acquire and utilize information, hindering their clinical work efficiency [20]. Research found that only a very small number of nurses use medical subject headings for retrieval, and most nurses did not realize that they could use medical subject headings for more efficient retrieval, which greatly restricted the use of information, especially in emergency departments where knowledge was constantly updated [21]. A survey among Canadian nurses showed limited mastery of information literacy skills in their professional practice [22]. An online survey investigating new graduates found that nurses used library resources least frequently in contrast to the Internet and websites [22]. And Zhang Na et al. [23] found that education level, income level, residence, and occupation were the influencing factors of information literacy.

Self-efficacy refers to the speculation and judgment of individuals on whether they are able to complete a certain behavior and the degree of confidence that people can use their skills to complete the work [24, 25]. Research showed that individuals’ information literacy self-efficacy was closely related to self-efficacy [26], which refers to self-confidence in information acquisition, analysis, processing, and utilization [27]. Therefore, we speculate that self-efficacy is an important factor affecting information literacy and consider it as an intrinsic factor in the Person-Context interaction theory.

Social support refers to the support from all social sources, including relatives, friends, and colleagues, giving individual spiritual or material help in subjective and objective manner [28, 29]. Research showed that information capacity was also closely related to social support [30]. Individuals with good social support have more ways to obtain information and access more information resources. Emergency department is a high-pressure and busy department, and good social support could help improve the professional abilities of emergency nursing staff [31]. Therefore, based on the Person-Context interaction theory, we speculate that social support is an external factor affecting the information literacy of emergency nurses.

## Aim of the study

So far, little is known about the level of information literacy among emergency department nurses. And a common drawback of existing research on information literacy among clinical nurses was that it was based on the assumption of population homogeneity and focused on explaining relationships between variables of interest in demographic information [32]. Given the crucial role of information in the context of emergency departments, it becomes imperative to enhance the information literacy level of emergency department nursing staff and investigate whether there exist different information literacy clusters in emergency department nurses.

Building upon prior studies, we seek to go a step further to identify different subtypes of information literacy among emergency department nurses in China and investigate the characteristics of the different subtypes to improve their working ability and efficiency. Based on the Person-Context interaction theory, the hypotheses of our study were as follows: **(a)** there are different subtypes of information literacy among emergency department nurses, **(b)** sociodemographic characteristics are the predictors of nurses’ information literacy and vary across the subgroups, and **(c)** self-efficacy and social support are the predictors of nurses’ information literacy and level of self-efficacy and social support of the subtypes are different among the subgroups.

## Methods

### Design

This was a multicenter, cross-sectional descriptive study to investigate the information literacy and its predictive factors among nurses from 65 emergency departments in Sichuan, Shenyang. Shaanxi, Shanxi, Beijing, Zhejiang, Chongqing, Guangxi, and Hainan. A self-reported questionnaire was utilized, consisting of the demographic questionnaire, nurse information literacy scale, self-efficacy scale and social support scale.

### Participants

From April to June 2023, 2490 emergency department nurses were selected in our investigation from a multicenter institution in China, covering 9 regions and 65 emergency departments. The inclusion criteria involved the emergency department nurses who had obtained the nurse qualification certificate and engaged in emergency work; the exclusion criteria included the nurses unwilling to participate in the investigation or not on duty during the investigation. Prior to distribution, we obtained informed consent from hospitals and enlisted the assistance of head nurses to administer the questionnaire via email. The questionnaire explained the purpose of our study and asked for their electronic written consent before conducting the investigation, and obtained informed consent signatures from all participants. Throughout the investigation, participants were informed of their right to withdraw at any time.

### Sample size

The sample size was calculated from 10 times the item under test (Li et al., 2018). There were 61 items in this questionnaire. Therefore, the calculation formula of sample size was N = (11 + 30 + 10 + 10) * 10 = 610, which mean that at least 610 subjects were required for this study. At the same time, considering the sample loss rate of 20%, the sample size should be further expanded. Therefore, the minimum sample size required was *N* = 610÷(1–20%) ≈ 763.

### Data collection

The researchers contacted the managers of each hospital and sent the questionnaires by email with the help of the head nurses in emergency department. When the questionnaires were sent out, the participants were given the same guidance. A total of 2490 questionnaires were distributed Supplementary Material  1, and 2384 valid questionnaires were collected, with an effective response rate of 95.74%. Among the 2490 participants, 48 nurses withdrew from the study; 33 questionnaires were partially filled; and there were 25 questionnaires with high consistency, filling the same response number for all items, and were regarded as invalid questionnaires.

### Questionnaire

#### The demographic questionnaire

The demographic questionnaire of emergency department nurses includes 11 items: age, working years, education background, title, residence, marital status, studying in spare time, using a database to search literature, number of recent medical literature readings and participation in information literacy training.

#### Nurse information literacy scale

Based on the Wadson’s [22] research on nurses’ information literacy questionnaire and an extensive review of literature, we translated and adapted the information literacy scale to better suit Chinese clinical nurses. Firstly, the original scale was independently translated by a university English teacher and a PhD in nursing management research. Subsequently, the researchers synthesized the two translations and addressed any ambiguities through adjustments. Next, we engaged the expertise of two university English teachers without professional backgrounds to translate the translated scale back into English. Finally, we conducted cultural adaptation, evaluated the content of the back-translation scale, and adjusted the expression of the translation scale. It was a self-designed questionnaire with 30 items, including 5 dimensions: information awareness (8 items), information knowledge (6 items), information ability (4 items), information ethics (6 items), and information support (6 items). The scoring method used Likert 5 points, and the higher the score, the higher the information literacy level. Before the formal survey, experts rated the items, and the scale had good content validity. We conducted a pre-survey among emergency department nurses, and the scale had good reliability and validity. The Cronbach’s alpha coefficient of the scale was 0.931. The explanatory variance of the 5 factors of the nurse information literacy scale was 67.229%. The internal consistency, split-half reliability and test-retest reliability were 0.878, 0.903 and 0.881 respectively.

#### Self-efficacy scale

The self-efficacy scale was compiled by Schwarzer et al. [33]. And the Chinese version was translated and revised by Wang Caikang [34]. The scale has 10 items and is widely used in China with good reliability and validity. In the process of answering, 1 ~ 4 points are given respectively from ‘completely disagree’ to ‘completely agree. In our study, Cronbach’s alpha coefficient was 0.938.

#### Social support scale

The social support scale was compiled by Xiao Shuiyuan [35]. It has 10 items, including 3 dimensions: subjective support (4 items), objective support (3 items), and utilization degree of social support (3 items). Items 1 ~ 4 and 8 ~ 10 are scored from 1 to 4 points in the order of options. Item 5 is scored from 1 ~ 4 points from ‘none’ to ‘full support. Items 6 and 7 are multiple topics where each option selected is counted as 1 point. In our study, Cronbach’s alpha coefficient of this questionnaire was 0.911.

### Data analysis

We used SPSS 26.0 statistical software and Mplus 8.3 for statistical analysis. The enumeration data were expressed by the number and percentage, and the measurement data were expressed in the form of mean ± standard deviation. The Chi-square test and variance analysis were used to screen statistically significant indicators. Logistic regression analysis was used to evaluate the influencing factors of potential categories. The data for information literacy were entered into the latent profile analysis. The models of different classes represent the number of information literacy categories for emergency department nursing staff, with one class initially and additional classes added incrementally until a unique solution could not be determined with maximum likelihood methods. Starting from a single model category, the number of model categories increased successively. The latent profile analysis model evaluation indicators include the Akaike information criterion (AIC); Bayesian information criterion (BIC); sample-size-adjusted BIC (aBIC); Lo-Mendell-Rubin (LMR) adjusted likelihood ratio test; Vuong-Lo-Mendell-Rubin likelihood ratio test (VLMR); Bootstrapped likelihood ratio test (BLRT), and Entropy. The smaller the AIC, BIC, and aBIC values, the better the model fitting. A higher Entropy value indicates a more accurate classification of the model. LMR and BLRT are often used in the model comparison, and *P*-value significantly indicates that K model categories are better than K-1 model categories. A low *P*-value indicates that the K-class model fits the data better than the K-1-class model [36]. Finally, according to the relevant results of all models, the best fitting models were selected by comprehensive evaluation of the above indexes, and the information literacy of clinical nurses was divided into different categories. All tests were performed using a two-sided approach, with a significance level of 0.05.

### Ethics approval

Our research was guided by the Declaration of Helsinki for ethical standards [37]. This research was approved by Air Force Medical University’s ethics committee (Number KY20224143-1).

## Results

### Emergency department nurses’ characteristics

In this study, a total of 2490 emergency department nurses were investigated and 2384 valid questionnaires were collected. The average age of the respondents was 30.00 years old (*SD* = 4.39; ranged from 20 to 40 years old), and the average years of working was 7.62 years (*SD* = 4.71; ranged 1 year to 22 years). 1031 nurses were from tertiary hospitals, 1147 nurses from secondary hospitals, and 206 nurses from primary hospitals. Among them, 980 had junior college degrees and 1404 had bachelor’s degrees or above. Other demographic information was shown in Table 1.

**Table 1:** Participants’ demography characteristics

Variable	Number	Proportion (%)
Age		
≤ 25	400	16.78
26–30	985	41.32
31–35	699	29.32
> 35	300	12.58
Years of work		
≤ 2	360	15.10
3–5	532	22.32
6–10	918	38.51
> 10	574	24.08
Educational level		
Junior college	980	41.11
Undergraduate or above	1404	58.89
Place of residence		
City	1639	68.75
Countryside	745	31.25
Marital status		
Single	645	27.06
Married	1719	72.11
Widowed or separated	20	0.84
Hospital-grade		
Tertiary hospital	1031	43.25
Secondary hospital	1147	48.11
Primary hospital	206	8.64
Title		
Nurse	794	33.31
Senior nurse	1219	51.13
Nurses-in-charge or above	371	15.56

### Descriptive statistics and correlations

Nurses’ information literacy was positively related to self-efficacy and social support. The descriptive statistics and correlations between emergency department nurses’ information literacy, self-efficacy, and social support were shown in Table 2.

**Table 2:** Descriptive statistics and correlations between information literacy, self-efficacy, and social support

Scale	M	SD	1	2	3	4	5	6	7	8	9	10	11
1Information literacy	65.86	17.68	1										
2Information awareness	13.02	5.01	0.706^**^	1									
3Information knowledge	16.73	5.10	0.793^**^	0.334^*^	1								
4Information ability	10.36	3.23	0.841^**^	0.419^**^	0.809^*^	1							
5Information ethics	11.75	4.32	0.800^**^	0.535^**^	0.476^**^	0.582^**^	1						
6Information support	14.01	4.91	0.799^**^	0.427^**^	0.525^**^	0.592^**^	0.576^**^	1					
7Self-efficacy	25.50	6.67	0.238^**^	0.185^**^	0.158^**^	0.212^**^	0.179^**^	0.209^**^	1				
8Social support	43.64	8.29	0.288^**^	0.267^**^	0.173^**^	0.202^**^	0.208^**^	0.270^**^	0.232^**^	1			
9Subjective support	11.72	2.52	0.217^**^	0.170^**^	0.157^**^	0.164^**^	0.159^**^	0.199^**^	0.207^**^	0.703^**^	1		
10Objective support	23.44	5.91	0.248^**^	0.240^**^	0.141^**^	0.169^**^	0.171^**^	0.239^**^	0.200^**^	0.921^**^	0.442^**^	1	
11Utilization of social support	8.48	1.88	0.201^*^	0.195^**^	0.111^**^	0.141^**^	0.163^**^	0.172^**^	0.117^**^	0.575^**^	0.372^**^	0.330^**^	1

Note: ^**^P < 0.01, ^*^P < 0.05

### Latent profile analysis

#### Exploratory latent profile analysis

**Fig. 1:**
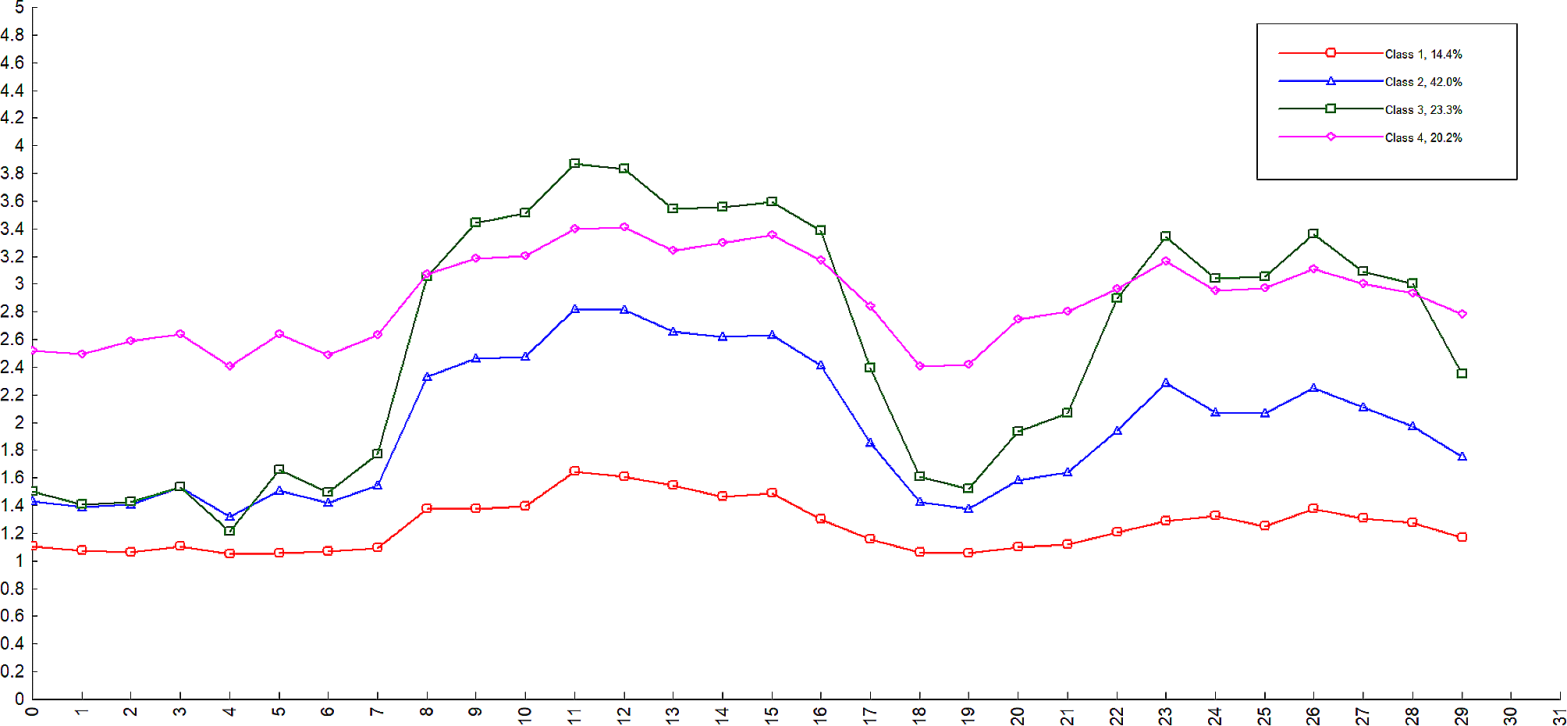
The different levels of information literacy among clinical nurses. Class 1: Low information literacy; Class 2: Moderate information literacy; Class 3: High information knowledge and support; Class 4: High information literacy

The best fitting latent profile analysis was the four-class model, which had the lower AIC (156359.659), BIC (157243.469), and aBIC (156757.355). The *P*-values of the LMR test (< 0.001), VLMR (< 0.001), and BLRT (< 0.001) were the smallest, suggesting that this model was statistically significant at the α = 0.05 level. In the four-class model, the information literacy of emergency department nurses was divided into 4 categories [38]. According to the analysis of 4 latent category characteristics, it could be categorized into relatively low (Class 1), medium (Class 2), and high (Class 4) information literacy levels, and high information knowledge and support (Class 3). The proportions of Classes 1, 2, 3, and 4 were 20.14%, 42.11%, 23.36%, and 14.39% respectively. The results were shown in Table 3; Fig. 1.

**Table 3:** Model fit indexes of latent profile analysis (*N* = 2384)

Model	K	AIC	BIC	aBIC	Entropy	LMR	VLMR	BLRT	Category probability
One-profile	60	188071.901	188418.493	188227.860	-	-		-	-
Two-profile	91	168275.691	168801.356	168512.229	0.940	0.000	0.000	0.000	50.21/49.79
Three-profile	122	160889.683	161594.420	161206.800	0.937	0.000	0.000	0.000	35.49/41.94/22.57
**Four-profile**	**153**	**156359.659**	**157243.469**	**156757.355**	**0.935**	**0.000**	**0.000**	**0.000**	**20.14/42.11/23.36/14.39**
Five-profile	184	152955.369	154018.251	153433.644	0.936	0.013	0.013	0.000	29.11/14.44/20.09/21.64/14.72
Six-profile	215	150460.692	151702.647	151019.546	0.945	0.016	0.016	0.000	14.01/ 28.69/18.92/13.63/13.21/11.54

Note: AIC: Akaike information criterion, BIC: Bayesian information criterion, ABIC: same-size adjusted Bayesian information criterion, LMR: Lo-Mendell-Rubin likelihood ratio test, VLMR: Vuong-Lo-Mendell-Rubin likelihood ratio test, BLRT: Bootstrapped likelihood ratio test

The average latent class probabilities for most likely latent class membership by latent class showed that Class 1, 2, 3 and 4 accounted for 97.4%, 96.7%, 94.8% and 96.7% respectively. The results were shown in Supplementary Table 1.

#### Information literacy of clinical nurses in different categories

The analysis of variance of the total score and five dimensions of information literacy in each group was statistically significant (*P* < 0.001). Further analysis of Least Significant Difference (LSD) showed that C1 < C2 < C3 < C4 in information literature, information consistency and information ethics; C1 < C2 < C4 < C3 in information knowledge and information capability; C1 < C2 < C3 and C1 < C2 < C4 in information support, and the difference between groups was statistically significant (*P* < 0.001). The results were shown in Table 4.

**Table 4:** Information literacy of clinical nurses in different categories

	N	Information literacy	Information consciousness	Information knowledge	Information capability	Information ethics	Information support
C1: Low information literacy	480	37.39 ± 6.19	8.60 ± 1.30	8.92 ± 2.98	5.41 ± 1.74	6.80 ± 1.49	7.67 ± 2.79
C2: Moderate information literacy	1004	59.06 ± 6.45	11.53 ± 3.30	15.56 ± 3.10	9.50 ± 1.87	10.25 ± 2.79	12.22 ± 3.33
C3: High information knowledge and support	557	77.55 ± 7.77	12.03 ± 3.17	21.28 ± 3.47	12.97 ± 2.29	13.39 ± 3.73	17.89 ± 3.64
C4: High information literacy	343	86.85 ± 9.64	20.42 ± 3.80	19.50 ± 3.25	12.65 ± 2.02	16.52 ± 3.28	17.76 ± 3.00
*F*		3653.621	1183.266	1220.438	1304.243	853.927	1003.423
*P*		0.000	0.000	0.000	0.000	0.000	0.000
LSD		C1 < C2 < C3 < C4	C1 < C2 < C3 < C4	C1 < C2 < C4 < C3	C1 < C2 < C4 < C3	C1 < C2 < C3 < C4	C1 < C2 < C3 C1 < C2 < C4

#### Characteristics of latent profile membership

Univariate analysis showed that the significant differences of the 4 groups were in age, years of work, educational level, title, hospital-grade, professional knowledge acquired in spare time, use of database for literature search, number of medical literature reading in the past month and participating in information literacy training (*P* < 0.05). There were significant differences in the self-efficacy and social support among the 4 groups (*P* < 0.05) and Class 4 showed the highest level of self-efficacy and social support. The results were shown in Table 5.

**Table 5:** The differences in nurses’ information literacy latent profiles in demography, self-efficacy and social support (*N* = 2384)

Variable	Respondents	Low information literacy	Moderate information literacy	High information knowledge and support	High information literacy	χ^2^/F	*P*
Age							
≤ 25	400(16.78%)	87(18.13%)	202(20.12%)	53(9.52%)	58(16.91%)	34.274	0.000
26–30	985(41.32%)	192(40.00%)	405(40.34%)	241(43.27%)	147(42.86%)		
31–35	699(29.32%)	139(28.96%)	289(28.78%)	181(32.50%)	90(26.24%)		
> 35	300(12.58%)	62(12.92%)	108(10.76%)	82(14.72%)	48(13.99%)		
Years of work							
≤ 2	360(15.10%)	80(16.67%)	173(17.23%)	55(9.87%)	52(15.16%)	31.045	0.000
3–5	532(22.32%)	96(20.00%)	249(24.80%)	112(20.11%)	75(21.87%)		
6–10	918(38.51%)	194(40.42%)	360(35.86%)	243(43.63%)	121(35.28%)		
> 10	574(24.08%)	110(22.92%)	222(22.11%)	147(26.39%)	95(27.70%)		
Educational level							
Junior college	980(41.11%)	237(49.38%)	393(39.14%)	201(36.09%)	149(43.44%)	24.873	0.000
Undergraduate or above	1404(58.89%)	243(50.63%)	611(60.86%)	356(63.91%)	194(56.56%)		
Place of residence							
City	1639(68.75%)	310(64.58%)	691(68.82%)	391(70.20%)	247(72.01%)	6.123	0.106
Countryside	745(31.25%)	170(35.42%)	313(31.18%)	166(29.80%)	96(27.99%)		
Marital status							
Single	645(27.06%)	134(27.92%)	292(29.08%)	126(22.62%)	93(27.11%)	16.586	0.011
Married	1719(72.11%)	338(70.42%)	707(70.42%)	429(77.02%)	245(71.43%)		
Widowed or separated	20(0.84%)	8(1.67%)	5(0.50%)	2(0.36%)	5(1.46%)		
Hospital-grade							
Tertiary hospital	1031(43.25%)	194(40.42%)	470(46.81%)	226(40.57%)	141(41.11%)	51.293	0.000
Secondary hospital	1147(48.11%)	216(45.00%)	470(46.81%)	270(48.47%)	191(55.69%)		
Primary hospital	206(8.64%)	70(14.58%)	64(6.37%)	61(10.95%)	11(3.21%)		
Title							
Nurse	794(33.31%)	192(40.00%)	362(36.06%)	131(23.52%)	109(31.78%)	42.656	0.000
Senior nurse	1219(51.13%)	230(47.92%)	485(48.31%)	327(58.71%)	177(51.60%)		
Nurses-in-charge or above	371(15.56%)	58(12.09%)	157(15.64%)	99(17.77%)	57(16.62%)		
Professional knowledge acquired in spare time							
None	66(2.77%)	37(7.71%)	9(0.90%)	17(3.05%)	3(0.87%)	153.017	0.000
Occasionally	1046(43.88%)	236(49.17%)	447(44.52%)	238(42.73%)	125(36.44%)		
When required by work	937(39.30%)	176(36.67%)	380(37.85%)	258(46.32%)	123(35.86%)		
Most of the time	335(14.05%)	31(6.46%)	168(16.73%)	44(7.90%)	92(26.82%)		
Utilization of databases							
No	328(13.76%)	88(18.33%)	90(8.96%)	118(21.18%)	32(9.33%)	151.137	0.000
Not really	1263(52.98%)	281(58.54%)	536(53.39%)	316(56.73%)	130(37.90%)		
Yes	793(33.26%)	111(23.13%)	378(37.65%)	123(22.08%)	181(52.77%)		
Number of medical literature reading in the past month							
None	1170(49.08%)	296(61.67%)	428(42.63%)	339(60.86%)	107(31.20%)	126.144	0.000
1–3	1013(42.49%)	158(32.92%)	482(48.01%)	185(33.21%)	188(54.81%)		
> 3	201(8.43%)	26(5.42%)	94(9.36%)	33(5.92%)	48(13.99%)		
Participation in information literacy training							
Yes	654(27.43%)	86(17.92%)	314(31.27%)	95(17.06%)	159(46.36%)	128.641	0.000
No	1109(46.52%)	238(49.58%)	439(43.73%)	317(56.91%)	115(33.53%)		
Unclear	621(26.05%)	156(32.50%)	251(25.00%)	145(26.03%)	69(20.12%)		
Self-efficacy	25.50 ± 6.67	23.41 ± 4.87	24.48 ± 5.04	26.24 ± 6.30	27.95 ± 10.19	41.417	0.000
Social support	43.64 ± 8.29	40.00 ± 7.96	42.56 ± 7.80	44.71 ± 7.90	47.39 ± 8.40	68.453	0.000
Objective support	10.72 ± 3.60	9.60 ± 3.22	10.44 ± 3.32	11.02 ± 3.54	11.87 ± 4.17	31.887	0.000
Subjective support	24.44 ± 4.94	22.56 ± 4.98	23.79 ± 4.79	24.99 ± 4.72	26.51 ± 4.68	54.014	0.000
Utilization of support	8.48 ± 1.88	7.84 ± 1.82	8.33 ± 1.77	8.70 ± 1.82	9.01 ± 2.05	35.190	0.000

#### Predictors of latent profile membership

Compared with Class 4, nurses with nursing titles and more than 6-year working experience were more likely to be grouped into Class 1; not reading literature in the past month was increased the probability of being grouped into Classes 1 and 2; the more years of work were were associated with a higher possibility of being grouped into Classes 2 and 3; the lower hospital-grade, in the countryside, less time spending in spare time to learn professional knowledge, less using databases to search literature, not participating in information literacy training, lower self-efficacy and social support were more likely to be grouped into Classes 1, 2 and 3. The results were shown in Table 6.

**Table 6:** The multifactor analysis of information literacy of clinical nurses by logistic regression (*N* = 2384). Note: bold: *P* < 0.05

Variable	C1 VS C4				C2 VS C4				C3 VS C4			
	β	OR	95%CI	*P*	β	OR	95%CI	*P*	β	OR	95%CI	*P*
Age												
≤25	-1.640	0.194	(0.094,0.401)	**0.000**	-1.763	0.171	(0.097.0.303)	**0.000**	-0.517	0.596	(0.388,0.916)	**0.018**
26–30	-1.451	0.234	(0.133,0.414)	**0.000**	-1.112	0.329	(0.214,0.505)	**0.000**	-0.595	0.552	(0.391,0.778)	**0.001**
31–35	-0.643	0.526	(0.335,0.826)	**0.005**	-0.433	0.649	(0.465,0.905)	**0.011**	-0.013	0.987	(0.758,1.284)	0.921
Years of work												
≤ 2	0.322	1.379	(0.720,2.643)	0.333	0.645	1.905	(1.148,3.163)	**0.013**	0.606	1.833	(1.255,2.676)	**0.002**
3–5	0.314	1.368	(0.800,2.340)	0.252	0.651	1.917	(1.281,2.868)	**0.002**	0.749	2.114	(1.544,2.896)	**0.000**
6–10	0.599	1.820	(1.197,2.768)	**0.005**	0.700	2.014	(1.468,2.761)	**0.000**	0.514	1.672	(1.301,2.148)	**0.000**
Educational level												
Junior college	0.284	1.328	(1.001,1.763)	0.051	-0.065	0.937	(0.752,1.166)	0.559	-0.166	0.847	(0.716,1.003)	0.054
Place of residence												
City	-0.401	0.670	(0.512,0.876)	**0.003**	-0.282	0.754	(0.610,0.931)	**0.009**	-0.268	0.765	(0.649,0.901)	**0.001**
Marital status												
Single	0.171	1.187	(0.414,3.404)	0.750	1.578	4.845	(1.524,15.410)	0.118	1.077	2.934	(1.395,6.171)	0.065
Married	0.790	2.204	(0.801,6.064)	0.126	1.919	6.816	(2.194,21.167)	0.201	1.402	4.063	(1.979,8.343)	0.067
Hospital-grade												
Tertiary hospital	-1.296	0.274	(0.173,0.434)	**0.000**	-1.197	0.302	(0.203,0.449)	**0.000**	-0.626	0.535	(0.376,0.762)	**0.001**
Secondary hospital	-1.662	0.190	(0.120,0.301)	**0.000**	-1.245	0.288	(0.193,0.429)	**0.000**	-0.894	0.409	(0.286,0.584)	**0.000**
Title												
Nurse	0.502	1.653	(1.002,2.725)	**0.049**	-0.065	0.937	(0.637,1.379)	0.743	0.292	1.339	(0.990,1.811)	0.058
Senior nurse	0.071	1.074	(0.714,1.614)	0.732	-0.004	0.996	(0.740,1.340)	0.978	-0.016	0.984	(0.775,1.250)	0.898
Professional knowledge acquiring in spare time												
None	1.995	7.354	(3.165,17.089)	**0.000**	1.167	3.214	(1.491,6.926)	**0.003**	0.083	1.087	(0.523,2.259)	0.823
Occasionally	1.050	2.858	(1.877,4.352)	**0.000**	0.785	2.193	(1.642,2.930)	**0.000**	0.369	1.446	(1.202,1.741)	**0.000**
When required by work	0.842	2.320	(1.515,3.554)	**0.000**	0.888	2.430	(1.822,3.240)	**0.000**	0.260	1.297	(1.076,1.564)	**0.006**
Using database to search literature												
No	0.667	1.949	(1.328,2.861)	**0.001**	1.086	2.963	(2.203,3.986)	**0.000**	0.077	1.080	(0.840,1.389)	0.547
Not really	0.817	2.265	(1.722,2.977)	**0.000**	0.962	2.617	(2.127,3.219)	**0.000**	0.548	1.729	(1.491,2.006)	**0.000**
Number of medical literature reading in the past month												
None	0.509	1.664	(1.027,2.695)	**0.039**	0.469	1.599	(1.125,2.272)	**0.009**	0.120	1.127	(0.883,1.439)	0.337
1–3	-0.150	0.861	(0.534,1.389)	0.539	-0.168	0.845	(0.601,1.188)	0.332	-0.061	0.941	(0.754,1.174)	0.589
Participation in information literacy training												
No	0.295	0.745	(0.565,0.981)	**0.036**	0.007	0.993	(0.795,1.241)	**0.043**	-0.009	0.991	(0.825,1.190)	**0.019**
Yes	-1.263	0.283	(0.205,0.390)	**0.000**	-1.186	0.305	(0.238,0.391)	**0.000**	-0.541	0.582	(0.487,0.969)	**0.000**
Self-efficacy	-0.060	0.942	(0.926,0.957)	**0.000**	-0.044	0.957	(0.945,0.969)	**0.000**	-0.016	0.984	(0.975,0.993)	**0.001**
Social support	-0.111	0.895	(0.881,0.909)	**0.000**	-0.081	0.922	(0.911,0.933)	**0.000**	-0.040	0.961	(0.952,0.970)	**0.000**

## Discussion

### Patterns of information literacy among emergency department nurses

Information literacy is essential throughout the entire cycle of emergency department nurses’ clinical nursing work, enabling them work effectively in an increasingly information-intensive emergency environment [39]. Therefore, current study on the latent profile analysis is valuable for hospital managers in understanding the different types of emergency department nurses’ information literacy, providing guidance to improve nurses’ information literacy levels. Based on the latent profile analysis, our study discovered substantial differences in information literacy among emergency department nurses, thus confirming **the hypothesis (a)**. The different categories of information literacy, were named as follows: low information literacy (Class 1), moderate information literacy (Class 2), high information knowledge and support (Class 3), and high information literacy (Class 4). The proportion of these categories were 20.14%, 42.11%, 23.36%, and 14.39%, respectively. Class 1 had the lowest level of information literacy, which indicates a major concern that should be focused on them. Class 2, accounting for the majority of the sample, had moderate information literacy, which could be the most common type of emergency department nurses’ information literacy in China. A systematic review conducted in 2017 ~ 2019 identified a general lack of informatics competencies [40] which was consistent with most types (Class 1 and Class 2) in our study. Emergency nursing work is dangerous and challenging, nurses need to have information literacy in the treatment of patients with critical clinical conditions particularly [41–43].

However, as the graph in the four-class model showed, the scores for the dimensions of information awareness, information ability, and information ethics were much lower than the other dimension scores in each subgroup. The possible reason might be that although emergency department nurses undertake heavy nursing work and have a lot of information needs, they may lack proficiency in actively collecting, sorting, or using information resources [44, 45]. Many nurses believe that clinical experience is more accurate than information evidence and have insufficient awareness of information literacy [46, 47]. The low information capability of emergency department nurses is mainly due to the lack of information acquisition ability, literature evaluation ability, and clinical decision-making ability [48]. They rarely or even do not use the database to retrieve literature [49, 50]. And the low levels of nurses’ information ethics are mainly due to the lack of understanding of intellectual property rights, laws, and policies [51, 52].

### Emergency department nurses’ information literacy predictors

The sociodemographic characteristics were different among the subgroups, confirming **the hypothesis (b)**. We have found that emergency department nurses in Class 1 were younger and had lower educational level and the proportion of primary professional titles was higher than other groups. Nurses who can’t use the database was larger for they spending less time on learning and the proportion of nurses who use the Internet and database was very small in Class 1. Research found that social media was an important way for nurses to improve their information literacy [53, 54]. An American study conducted among 349 nurses found their most common daily electronic sources of information were electronic medical records which may contain links to external sources (72%), followed by general search engines daily (39%), then websites with medical information (23%) in hospital [55]. Moreover, without digital source evaluation skills, nurses were unable to discern reliable online sources effectively [56, 57]. Class 2 and Class 4 were referred to as the ‘moderate information literacy’ and ‘high information literacy’ subtype, and both showed a similar pattern for the five dimensions of information literacy. Class 2 showed a medium level for all information literacy dimensions. The majority of emergency department nurses in the two classes shown the characteristics of spending more spare time acquiring professional knowledge, using databases to search the literature, and reading medical literature. Class 3 showed the highest score in the information knowledge and support dimensions. This type of demography was characterized by older age, longer working years, higher educational level, and professional titles.

Based on the Person-Context interaction theory [58], our further study found that self-efficacy and social support were closely related to the information literacy of emergency department nurses, and were different among the subgroups, which were the predictive factors of information literacy, confirming **the hypothesis (c)**. The Person-Context interaction theory holds that individuals and situations interact with each other. Emergency nurses operate within a demanding emergency environment that necessitates proficient information skills. Their information literacy is shaped by a combination of internal and external factors. Therefore, we opted to investigate the self-efficacy as an internal factor and social support as an external factor to explore their impact on the information literacy of emergency nurses and further analyze their differences in different types of information literacy. Compared with Class 4, lower self-efficacy and social support had a higher likelihood of belonging to Class 1. Research showed that a good sense of self-efficacy helped individuals improve their self-confidence [59, 60]. Therefore, emergency nurses with high self-efficacy can better analyze and process clinical information, and with good information literacy. Research showed that under high emergency pressure, social support could alleviate their fatigue and stress [61]. The emergency center is the department with the concentration of critically ill patients and the most types of diseases, therefore good social support can provide subjective and objective assistance to emergency nurses, broaden their information acquisition, and provide more information resources in the form of providing information training, continuing education, and information support, which is conducive to the improvement of their information literacy.

### Relevance to clinical emergency practice

In the context of information medicine, the level of emergency nurses’ information literacy is low, posing challenges in meeting the demands of information nursing development [62, 63]. The complexity of diseases in the emergency department require information technology in medical equipment and the development of information capabilities among medical personnel [64]. The capability of using information is essential for emergency nurses to adapt to emergency environments, allowing them to quickly solve clinical problems and critical issues [65]. To align with the advancements in intelligent medicine, it is imperative to improve the information literacy of emergency department nurses. Through potential profile analysis, we found that the information literacy of emergency department nurses fell into 4 categories, and further explored the predictive factors of different categories. These predictive factors are of great significance to improve their information literacy of emergency department nurses across different latent profile memberships through targeted interventions.

It suggests that emergency department managers utilize and consider these characteristics to improve emergency nurses’ information literacy. Low information literacy emergency nurses require greater attention compared to their high information literacy counterparts. Nursing experience of more than 6 years, nurse title, not reading the literature in the past month, lower hospital grade, rural areas, less time spent in spare time to learn professional knowledge, low utilization of databases to search the literature, and not participating in information literacy training have a higher likelihood of belonging to Class 1. It implicates that more attention needs to be paid to these factors to improve nurses’ information literacy levels. Our research was consistent with Nowrouzi’s [66] findings that highly educated nurses had better work ability, so they could master information skills and had a high level of information literacy. Therefore, improving the education level of nursing staff and strengthening their continuing education is very important.

Simultaneously, emergency nurse managers should focus on both the initiative and self-efficacy of emergency department nurses as well as leverage the power of social support, giving full play to the role of internal and external factors in clinical practice. Head nurses in emergency department should regularly encourage nurses to boost their confidence and enhance their self-efficacy. Hospital managers could support their participation in learning and training programs, especially in the field of information. Additionally, they can incentivize and provide support by evaluating and rewarding their information skills, thus fostering the improvement of their information literacy. Family members of emergency nurses should also fully understand and support their work, and pay more attention to their needs.

### Limitations

There are some limitations in our study. First of all, due to the cross-sectional nature of the data, we cannot draw definite conclusions about the directionality of associations between the identified profiles and covariates. Secondly, our study was conducted in the form of a self-report questionnaire and the results tended to be subjective. Thirdly, the main force in Chinese emergency department is young nurses, which leads to age homogeneity. So external validity may be limited to nurses of different age structures in other countries.

## Conclusion

In conclusion, through the use of latent profile analysis, we have identified four distinct categories of information literacy among emergency department nurses: ‘Low information literacy (Class 1)’, ‘Moderate information knowledge (Class 2)’, ‘High information knowledge and support (Class 3)’ and ‘High information literacy (Class 4)’. We have also determined several factors that influenced the classification of information literacy among these nurses. These predictors include age, working years, title, hospital grade, place of residence, literature reading, database use, time spent in spare time to learn professional knowledge, information literacy training, self-efficacy, and social support.

### Electronic supplementary material

Below is the link to the electronic supplementary material.

**Supplementary Material 1**: Questionnaire

**Supplementary Material 2**: Table of Average Latent Class Probabilities for Most Likely Latent Class Membership

## Data Availability

The datasets generated and analyzed during the current study are not publicly available due to the protection of the privacy of consulting experts but are available from the corresponding author (906963251@qq.com) on reasonable request.
