# Supplementary material for: Patterns of information literacy and their predictors among emergency department nurses: a latent profile analysis based on the person-context interaction theory
Source: BMC Nurs. 2024 Jan 26;23:71. doi: 10.1186/s12912-024-01756-9 (PMC10811938; doi:10.1186/s12912-024-01756-9)
Supplement: Supplementary file 1 — Supplementary Material 1: Questionnaire [file 12912_2024_1756_MOESM1_ESM.docx]

**Supplementary material**

The questionnaire and scales: https://www.wjx.cn/m/79503496.aspx
