# Supplementary material for: Patterns of information literacy and their predictors among emergency department nurses: a latent profile analysis based on the person-context interaction theory
Source: BMC Nurs. 2024 Jan 26;23:71. doi: 10.1186/s12912-024-01756-9 (PMC10811938; doi:10.1186/s12912-024-01756-9)
Supplement: Supplementary file 2 — Supplementary Material 2: Table of Average Latent Class Probabilities for Most Likely Latent Class Membership [file 12912_2024_1756_MOESM2_ESM.docx]

**Supplementary Table 1** Average Latent Class Probabilities for Most Likely Latent Class Membership (Row)

by Latent Class (Column)

| Class | C1(%) | C2(%) | C3(%) | C4(%) |
| --- | --- | --- | --- | --- |
| C1 | **0.974** | 0.026 | 0.000 | 0.000 |
| C2 | 0.010 | **0.967** | 0.018 | 0.005 |
| C3 | 0.000 | 0.030 | **0.948** | 0.022 |
| C4 | 0.000 | 0.011 | 0.023 | **0.967** |
